# Factors affecting the self-efficacy of medical teachers during a health crisis – a qualitative study on the example of the COVID-19 pandemic

**DOI:** 10.1186/s12909-023-04393-z

**Published:** 2023-06-02

**Authors:** Magdalena Cerbin-Koczorowska, Piotr Przymuszała, Łucja Zielińska-Tomczak

**Affiliations:** 1 Department of Medical Education, Poznan University of Medical Sciences, 7 Rokietnicka St, 60-806 Poznan, Poland; 2 Edinburgh Medical School: Medical Education, University of Edinburgh, Chancellor’s Building, EH16 4SB Edinburgh, Scotland United Kingdom

**Keywords:** Medical teachers, Self-efficacy, Medical education, Online education, e-learning, COVID-19 pandemic

## Abstract

**Background:**

The diversity of tasks entrusted to medical teachers with their simultaneous responsibility for the safety of patients and the effective education of future healthcare professionals requires maintaining a skillful balance between their teaching, scientific and clinical activities. Meanwhile, the COVID-19 pandemic disrupted the work of both healthcare facilities and medical universities, forcing already overworked medical teachers to establish a new balance. One’s ability to perform effectively in new, ambiguous, or unpredictable situations was described by Albert Bandura as a self-efficacy concept. Consequently, this study aimed to identify factors affecting the self-efficacy of medical teachers and the influence of the COVID-19 pandemic on them.

**Methods:**

Twenty-five semi-structured interviews with medical teachers were conducted using a flexible thematic guide. They were transcribed and analyzed by two independent researchers (researcher triangulation) with phenomenology as the qualitative approach.

**Results:**

Identified themes demonstrate a process of the evolvement of clinical teachers’ self-efficacy in response to the sudden outbreak of the COVID-19 pandemic, namely the decline of self-efficacy in the first phase of the crisis, followed by building task-specific self-efficacy and the development of general self-efficacy.

**Conclusions:**

The study shows the significance of providing care and support for medical teachers during a health crisis. Crisis management decision-makers at educational and healthcare institutions should consider the different roles of medical teachers and the possibility of overburden associated with the cumulation of the excessive number of patient, didactic, and research duties. Moreover, faculty development initiatives and teamwork should become a vital part of the organizational culture of medical universities. A dedicated tool acknowledging the specificity and context of medical teachers’ work seems necessary to quantitatively evaluate their sense of self-efficacy.

## Background

The work of medical teachers can be compared to a three-legged stool, in which all three legs, namely teaching, scientific activity, and clinical practice, must be properly balanced [1]. However, keeping this balance can constitute a challenge and source of stress resulting from the simultaneous need to provide adequate care to patients, high expectations of the employer in terms of publishing effectiveness, and a sense of responsibility for the effective education of future medical professionals. The elevated levels of stress at work over a longer period of time may lead to burnout, which is defined by emotional exhaustion, depersonalization (negative and cynical approach toward other people), and increased inefficacy feelings [2], which in the case of medical teachers, who very often are simultaneously also active healthcare professionals, may negatively affect both patient safety and the quality of education of medical students. Meanwhile, studies show that healthcare professionals are especially susceptible to burnout, which may lead to very harmful effects, including substance abuse, depression, suicidal ideation, medical errors, reduced workers’ productivity, and lower patient satisfaction [3, 4].

Previous studies point to a link between the stress and burnout of teachers and the educational process’ effectiveness [5, 6]. Meanwhile, despite the influence of teacher’s well-being on the learning process climate, too little attention seems to be paid by medical schools to this topic and sustaining the social and emotional wellness of teachers [7]. The profession of a medical educator is strictly connected with intensive interpersonal interactions with, among others, their patients and students, which, however, may contribute to the development of burnout [8]. The diverse requirements and challenges associated with each of their three roles may also decrease medical teachers’ abilities to focus on students and their learning. For example, their clinical role may be connected with decreased autonomy and limited time for patients; the scientific role with increased pressure to write articles and obtain grants; and finally, the teacher role with administrative overload [9, 10]. Consequently, the perception of work in higher education institutions is also changing from once a low-stress to a high-occupational-stress environment [10]. It may have a negative effect on teachers’ health, contributing to reduced physical and psychological well-being as well as negatively impacting their family and private life [5]. Noteworthy, the price resulting from occupational stress and burnout is also paid by the institutions in the form of teachers’ turnover intentions [11], which seems especially important given the observed shortage of healthcare personnel, as well as other outcomes like worse interpersonal relations, and the reluctance of staff to assume additional tasks or implement innovations [5].

The situation might have been exacerbated by the COVID-19 pandemic, which disrupted the organization of healthcare systems worldwide and significantly increased the workload and stress of healthcare professionals’ work [12]. Moreover, due to the dynamic character of the situation, many educational institutions, including medical universities, suspended their traditional face-to-face classes and started to realize the education process online [13]. The novel and sudden character of this process could have constituted an additional burden and source of stress for medical teachers, especially given the limitations of online learning in realizing all learning outcomes in medical curricula [14–16]. This simultaneous increase in expectations towards medical teachers, both in the context of their clinical and educational duties, could have violated the aforementioned delicate balance of medical education in terms of the division of responsibilities between caring for the patients and teaching future healthcare professionals. As a result, providing medical services and hosting the education and training of future generations of health professionals seems to compete even more for limited time and resources.

The way in which people cope with such a new and threatening situation seems to depend on their belief in their efficacy and can range from fear and avoidance when this belief is low to a confident and active approach in situations they believe are within their capabilities [17]. This concept of self-efficacy described by Albert Bandura can take two forms, namely general and task-specific. The former is more holistic and relates to “*the belief in one’s capabilities to organize and execute the courses of action required to manage prospective situations*” [18]. In contrast, the latter is focused on the implementation of specific activities [18], for example, giving an online lecture for medical students. Teachers’ self-efficacy seems to have an important role in coping with stress, preserving mental health, and preventing occupational burnout [19, 20]. In his other works, Albert Bandura also elaborated on the sources of self-efficacy, identifying four of them: 1) enactive mastery experiences, which underline the upbuilding effect of successes and the deteriorating effect of failures on one’s self-efficacy as well as the possibility to treat overcoming obstacles as learning opportunities; 2) vicarious experiences allowing to draw from observation and comparison of one’s capabilities with other people; 3) verbal persuasion along with other social influence types representing the importance of positive feedback and the belief of significant others in one’s capabilities; and 4) physiological and affective states – emphasizing the effect of psychological and emotional state on the self-efficacy [21].

The unprecedented character of the COVID-19 pandemic makes it impossible to predict the exact mechanisms of its influence on medical teachers’ self-efficacy without obtaining new, up-to-date data. Meanwhile, the international scope of the crisis means that the problems faced by medical universities today may have global and unpredicted consequences in the future. Despite the presence of tools dedicated to the assessment of general and task-specific self-efficacy among Polish teachers, they predominately concern teachers of primary, secondary, and high schools [20]. However, there is a risk that they may not take into account the specificity of the educational environment in which medical teachers have to combine not only didactic and research activities but also numerous clinical duties associated with the responsibility for the health of patients.

Consequently, in this study, an attempt was made to answer the question: “How did the health crisis affect the beliefs of medical teachers about their self-efficacy?” and identify factors that may enhance or lower medical teachers’ sense of self-efficacy along with the way they were influenced by the crisis caused by the COVID-19 pandemic. In the study, it is also planned to compare the identified factors with those related to classroom teachers.

## Methods

### Researchers’ characteristic

The first author is a pharmacist with a Ph.D. degree. She also graduated the MSc Clinical Education Programme and has previous experience in qualitative research methods. The research team also included two other researchers with different backgrounds to avoid bias. The second author is a physician with research experience in both online learning and qualitative methodology. The third author is also a pharmacist holding an additional Bachelor’s in Public Health degree, which constituted a strength during data analysis and interpretation.

### Methodological design and the method

The phenomenological approach was chosen for this study as ‘*phenomenological approach is well suited to studying affective, emotional, and often intense human experiences’* [22]. It can be distinguished into two prominent approaches – descriptive (Husserlian) and interpretive (Heideggerian). While the first one assumes the ‘presuppositionlessness’ of the researcher, who should discover the objective reality as a tabula rasa, the second one notices the impossibility of fully eliminating the researcher bias and proposes the interaction between the interviewer and interviewee as a way to achieve more accurate experiences interpretation [23]. In view of the fact that the researchers are also academic teachers, the second (Heideggerian) approach was adopted in the study.

The study was conducted from June 2021 to March 2022 in the form of semi-structured interviews with teachers of Poznan University of Medical Sciences (PUMS). Inclusion criteria into the study group were as follows: the status of an academic teacher of PUMS, teaching practice-oriented subjects, and the consent to participate in the study. Apart from that, no restrictions for potential participants in regard to their gender, profession, or teaching experience were introduced, but relevant demographic information on these issues was collected to allow differentiating within the sample. We used convenience sampling. Invitations to participate were sent to prospective participants meeting inclusion criteria by e-mail. They informed potential respondents about the aims of the study, its scientific, anonymous, and voluntary character, and the possibility of resigning from participation at any moment. Due to the increased workload associated with the COVID-19 pandemic, which could have hindered the recruitment process, material incentives in the form of gift cards to a bookstore chain were planned for respondents to increase their willingness to participate in the study.

Due to the epidemiological safety issues, the interviews were conducted with the use of MS Teams. They started with an explanation of the study protocol to the respondent, answering any potential questions, obtaining informed consent for participation in the study, and recording its course. The interviews revolved around a flexible thematic guide, which was developed by the main author during the research planning phase. When compiling the list of questions, the authors used the factors listed in the existing tools for teachers’ self-efficacy [24–26]. Taking into account that these works were not carried out in the realities of higher education, the interview was semi-structured, allowing the emergence of new themes. Simultaneously, given the period in which the research was conducted, the interviewer also referred to the pre-pandemic time during the interview. The interviews’ outline is presented in Table 1. After every interview, the recordings and demographic data of the respondents were immediately encoded and further processed anonymously.

**Table 1:** Interview’s outline

1. Work experience as an academic teacher (opening question)
2. Reasons for becoming an academic teacher
3. Perceived difficulties of working as an academic teacher (including both arguments for and against)
4. Subjects taught and discussion on teaching before and during the pandemic
5. Organization of work in the workplace when the pandemic started
6. Emotions accompanying the beginning of the pandemic
7. Relations among co-workers during the pandemic (and in comparison before the pandemic)
8. Support received from the University at that time
9. The effect of the pandemic on other areas of work
10. The effect of the pandemic on self-efficacy in other roles
11. Other reflections (closing questions)

### Participants

Twenty-five medical teachers participated in the project, and each of the interviews lasted for about an hour (between 37 min and 1 h 15 min). The respondents represented various professions and had varied experience in the work of an academic teacher. The characteristics of the respondents are presented in Table 2.

**Table 2:** Respondents’ characteristics

Respondents’ number	Gender	Profession	Teaching experience
R1	female	Dentist	38 years
R2	male	Nurse	3 years
R3	female	Physiotherapist	10 years
R4	female	Physician	17 years
R5	female	Physician	22 years
R6	female	Midwife	9 years
R7	female	Physician	4 years
R8	female	Physician	14 years
R9	female	Physician	10 years
R10	female	Physician	5 years
R11‍	male	Pharmacist	6 years
‍R12	female	Physician	2 years
‍R13	female	Physician	5 years
‍R14	male	Pharmacist	10 years
‍R15	female	Physician	7 years
‍R16	male	Dentist	5 years
‍R17	female	Pharmacist	3 years
‍R18	female	Laboratory diagnostician	3 years
‍R19	female	Physician	10 years
‍R20	female	Nurse	17 years
R21‍	female	Physician	3 years
R22‍	male	Physician	3 years
R23‍	female	Physician	5 years
‍R24	female	Physician	4 years
‍R25	female	Pharmacist	1.5 year

### Data analysis process

After the interviews, the recordings were subjected to a literal transcription and subsequently analyzed by two researchers with phenomenology as the qualitative approach, who worked independently (researcher triangulation) in order to view the results from a broader perspective [27]. The data analysis process implemented the interpretative phenomenological analysis, in which the main goal is to understand people’s experiences [28]. The data analysis process involved an open-coding system and included stages described by Pietkiewicz and Smith [29], namely repeated reading and taking notes, transforming notes into emerging themes, then finding relationships and grouping the themes. The SRQR guidelines (standards for reporting qualitative research) were followed when reporting the study [30].

### Ethical issues

Prior to the study, its project was submitted to the Bioethical Committee of the Poznan University of Medical Sciences, which decided that its approval was not necessary under Polish law since the study was not a medical experiment and did not involve patients (Case number: KB-335/21). Still, efforts were made to ensure the highest ethical standards during the study. Participants were informed about its aims, course and data collection methods as well as assured about its anonymous and voluntary character. Furthermore, informed consent was obtained from all participants before the interviews, and they could resign from further participation at any stage of the study. As disclosed above, the authors also paid special attention to protecting the anonymity of collected data.

## Results

The most striking result to emerge from the data is the extent to which the COVID-19 pandemic affected various areas of the respondents’ work and how separate factors related to it seemed to intertwine among medical teachers’ diverse duties. The statements of the respondents demonstrate a process in which the first response to the emerging crisis was a rapid decline in their perception of their self-efficacy. This was followed by the subsequent rebuilding of task-specific self-efficacy and gradually maturing to a growing sense of general self-efficacy. The deterioration in self-efficacy in the first phase was aggravated by the specificity of the work of clinical teachers, resulting in a sense of fear and helplessness. Then, as time passed, the respondents built back their self-efficacy with a sense of responsibility for others, at first in minor tasks, aiming to maintain the continuity of the didactic process, and then, this process seemed to evolve and started to involve building a sense of self-efficacy not only for individual tasks but also their performed role. Among the many factors that affect self-efficacy at various stages of responding to the crisis, the ones that deserve special emphasis include the multifaceted nature of interpersonal relations and selected elements of organizational culture and institutional support—described in detail in dedicated sections below.

### The decline of self-efficacy in the first phase of the crisis

**Fig. 1:**
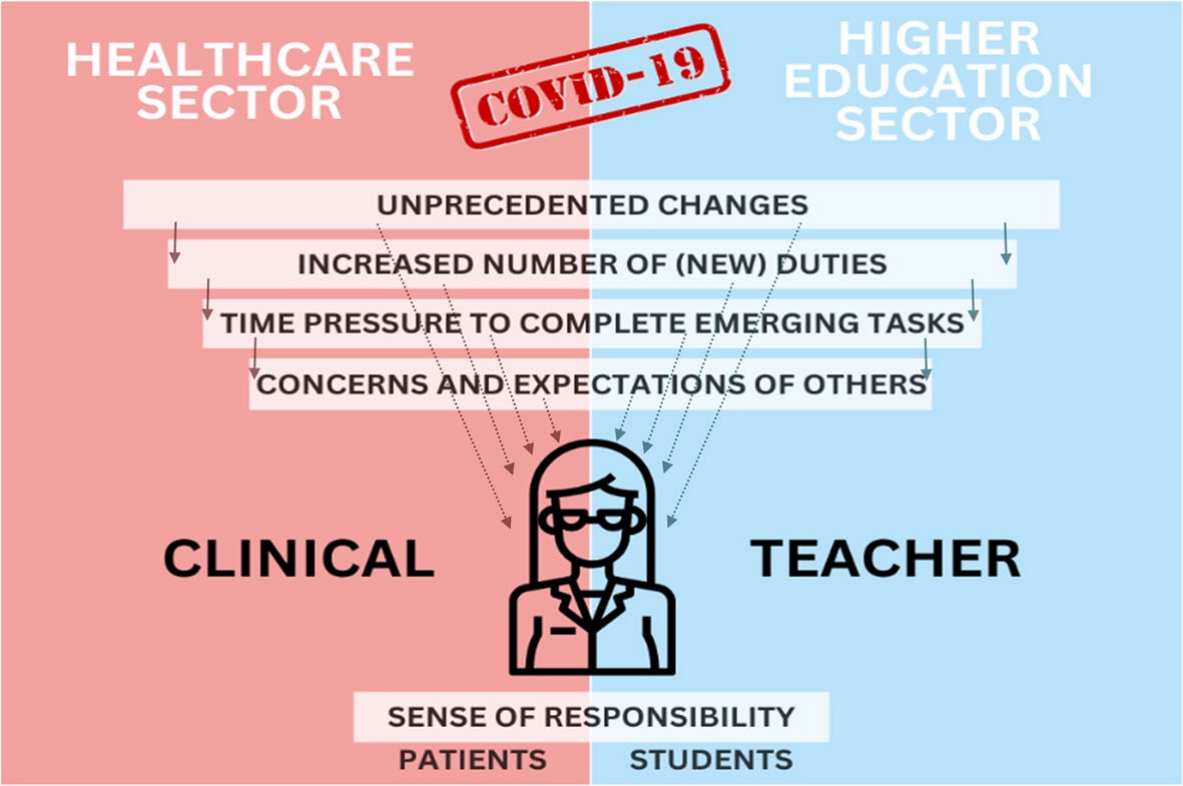
Factors indirectly (solid lines) and directly (dotted lines) affecting clinical teachers’ self-efficacy during the first phase of the health crisis

The pandemic significantly affected the everyday functioning of both the sector of higher education and the functioning of healthcare facilities. The everyday functioning of clinical teachers in both environments simultaneously multiplied the potential factors, directly and indirectly, influencing their sense of self-efficacy in both roles performed, as shown in Fig. 1.

The factor that most often appeared in the respondents' statements were unprecedented changes in the first phase of the crisis in response to a hitherto unknown threat and epidemiological precautions introduced both at hospitals and at the University that led to changes in their everyday functioning, structural and organizational redesigns of either of those environments. In the hospital setting, these involved the introduction of online consultations with patients, limited numbers of patient admissions, and the scope of performed procedures due to pandemic restrictions. At the same time, at the University, all classes with students were moved to distance learning forms, including the clinical and practical ones, since all wards and clinical hospitals were closed for students to prevent the spread of the virus.

The situation was additionally exacerbated by the frequency and unpredictability of pandemic restriction changes resulting in teachers’ sense of uncertainty and lack of stability. Teachers had limited opportunities to freely plan their classes as they saw fit, with a simultaneous constant need to monitor frequently changing recommendations and guidelines on the new virus, which at some point made it impossible to track them.

R2: *“I will say this: planning classes before a pandemic, I mean, it may sound strange, but it was much, much, much, much simpler. I just had a flow chart of things that I do, and simply by going through my classes, I was ticking them off—I showed, I did, I passed, I questioned. During a pandemic, it is not that simple anymore because I have to adapt to whether I will or will not be able to do something, so it is often the case that although I have something planned, I move it to later because, for example, we do not have the possibility and I will have to do it later when I have such an opportunity because the restrictions will be reduced […] so my plans this year were actually changing from week to week.”*

R24:* “These frequent changes, such as the ones introduced by the University, related to the didactic process and work-related imposed by the Director [of the hospital] were sometimes difficult because there were frequent changes. For example, in one week, the ward was completely closed to scheduled patients’ admissions and there were only acute admissions, then the scheduled ones were restored […] it was changing very often.”*

The changing recommendations and regulations were accompanied by an increased number of duties, a large part of which were completely new and significantly different from the specificity of the activities with which the respondents were familiarized so far. The number of patients needing help increased across various fields of medicine, exceeding the capacities of wards and healthcare personnel. Additionally, some respondents or their colleagues started to work at COVID-19 wards, which entailed further burden.

R4: *“We also had a huge burden related to the enormity of patients who suddenly needed help, so there was the question of the willingness and the time possible to devote.”*

R8: *“We, too, as clinicians, collided with a different reality in the clinic, and that was also something we had to learn a bit differently because we had to redesign one department. The work system in the ward was different. Some of us went to the COVID hospital, so our normal activities were also turned upside down, we had teleconsultations with patients at the clinic, not physical visits.”*

Apart from the clinical overload described above, their preparation for online classes also took significantly more time than before the pandemic. As a result, respondents experienced a sense of increasing pressure accompanied by a lack of time to deal with all emerging tasks. This seemed to negatively affect respondents’ sense of self-efficacy.

R4: *“the amount of work put in its preparation, coordination, the preparation of the [educational] material was enormous. It really was incomparable to the preparation of traditional classes, also absolutely much more work […], not all assistants wanted to be involved in it on an equal level.”*

This observation finds confirmation in the words of another respondent whose department, due to the shape of the class schedule, had more time to prepare for online classes, which gave them more comfort and a sense of relief.

R8: *“We probably even had such comfort in quotation marks that we had such a gap in diabetology that there were many classes in early September, October, and November, so they took place as normal. When the lockdown came, we did not have diabetology right away—it was sometime around April, so we had a certain comfort of time to sit down and work it out, and there really was, I was sitting with my boss, there was a lot of brainstorming what to do.”*

Similarly, once the materials for the classes were ready, they could be easily reused, which reduced the time necessary to prepare for subsequent editions of the classes for the following student groups.

R4: *“On the other hand, once these materials were prepared, in these subsequent courses, of course, we modified them a bit depending on what we saw worked or not, but then it was easier, of course, these next courses. So, the first year was harder. Later in the next year, I will be honest, it went absolutely faster.”*

Time management also constituted an important issue for respondents. Some of them noticed that they lacked the time and had to work extra hours to check and discuss students’ assignments.

R1: *“It was also difficult, especially since at the beginning, all this situation surprised us, and the students were coming up with that, for example, he could connect at 20:30. The first group—I played along a bit, but then I realized, no way, I am only connecting with someone all day, that’s it. […] later we introduced some, uh, no-freedom to arrange, but only within these hours of classes or together we agreed on a convenient date as the rest wanted.”*

Online learning might have also been perceived as more time-consuming by some teachers as it prevented them from multi-tasking and balancing their clinical and didactic duties.

R9:* “it deeply disorganized the work in the ward if the physician who was on the ward had to conduct online classes because, as I said, sometimes, unfortunately, we had to do five things at the same time. With students [at the ward], it was doable because some things can be done with students, somehow even involve some of them for help. And when you are online, then at some point there were such pathological situations that most doctors in the ward were locked in their offices conducting online classes and only one was left for the entire ward, which is really not cool.”*

R22: *“A negative aspect of distance learning is the fact that I am tied to a computer while conducting online classes. I have no or very limited possibility of taking care of my patients. When the classes are at the ward, I take the students to patients, we examine them together, talk with them, consider potential options. […] The realization of it while being on the other side of the camera is practically impossible. I’ve been thinking about various science-fiction ideas—to put a GoPro camera on my forehead, for example, so that students could see what is happening, but this creates very big problems related to the protection of personal data. The patients would have to agree to it, it is impossible to realize.”*

Finally, the work overload also affected the scientific activity of academic teachers.

*R4: „In the context of the NCN [Narodowe Centrum Nauki – National Centre of Science – a Polish grant agency], I had to write for an extension of the substantive report because I was not able to write a single article during the pandemic, and you know there are deadlines and various problems may arise from it because it was simply working at night.”*

Balancing these various roles and responsibilities came at the expense of respondents’ personal life.

R4:* “It all comes at the expense of private time. I can see it very clearly, because the biggest, so to speak, sensor are children who, for example, see that mum—you have no time recently or you are on the computer all the time, because my children indeed saw me all the time on Teams, online, because I basically worked non-stop, not to mention scientific work, which in all of this was probably only at night.”*

In addition, respondents were faced with uncertainty and fear as well as increasing concern and expectations on the part of both students and patients. This all led to a sense of frustration and crisis for some respondents. They wanted to help their patients better but felt that the situation was beyond their control. Patients’ and students’ frustration unrightfully directed against them enhanced this feeling of helplessness.

*R6: “We are just as helpless in the sense that there are certain rules imposed from above, and I personally feel that the patients also direct anger, helplessness, and frustration at us, the visits have been canceled. […] new regulations come out, and we are all victims, in fact, in this situation. Still, frustration and anger are directed towards healthcare workers with enormous strength, and I really feel it on a daily basis, and it is also very difficult.”*

Due to the specificity of the work performed, the sense of uncertainty was accompanied by a strong sense of responsibility for the people entrusted to their care.

R22: *“And when it comes to non-COVID patients, there is growing frustration about the fact that we cannot help patients. Our patients should also be admitted to us for scheduled diagnostics, but we are forbidden to admit such patients, and they are waiting, we don’t know for how long. And the only way for them to get to the hospital is that their health has to worsen enough to endanger their lives, and only then can they get to us for the diagnostics they might have had three months earlier.”*

R9*: “These [student] groups are also big, so they cannot enter the ward, and I think it is probably the worst moment, maybe not for me, for me, as an academic teacher, it is not only the regime that I will show them fewer patients, it is not something that cannot be coped with, because I will simply use words, but for them, as students, it is very difficult, and they complain about it very much. […] such helplessness that everything is not as it should be, and it is not even clear who to blame for it. Because, on the other hand, as a doctor, I care more about the health of patients, so I prefer to tell students something than, for example, to expose patients that they will get infected, so I understand a bit. I mean, I feel for the students, but even more, I feel for the patients.”*

Respondents reported that they found it more difficult to convey the practical or clinical aspects of the course. They emphasized that students cannot acquire practical skills without actually performing them or having contact with patients.

R24:* “I think that in such a normal situation, they have a better chance to learn the contact with the patient from me—I don’t know—that when we are going in, we say hello, the way we ask questions, the way we talk to these patients.”*

R21: *“Even the best presentations, […] or uploading videos to YouTube cannot provide the comfort I had when conducting classes with patients, for example with pneumonia, when they [students] could themselves hear pneumonia. Because we normally were going to patients […]. Once you hear it, you will remember it for the rest of your life. And when we play YouTube videos, despite our best intentions, we are not able to show it to them in any way. Or such skills as percussion, throat examination where you have to be able to quickly put that stick in the mouth of such a little child to open the mouth. This is a problem in the sense that we felt unsatisfied here, as a team, not only me but the whole team. How were we supposed to teach physical examination to our students online? It didn’t make sense.”*

In this context, teachers doubted whether students were able to gain intended learning outcomes, which for some of them, took the form of personal defeat.

R6: *“The assumed goals were not achieved, that is, the learning outcomes, neither in the field of communication, nor the field of social competencies, nor the field of physical examination […] They did not acquire these competencies. They just didn’t do it. […] this subject did not really take place in the sense that I discussed the materials online with them, and that was all, and they, well, the subject was not realized, in my opinion, it simply should be repeated.”*

They also questioned the reliability and credibility of their role as an assessor. Respondents pointed out that as teachers, by giving students credit, they confirm that they have certain qualifications. At that time, they emphasized the responsibility that rests on them, pointing to the relationship between making the wrong decision that a student passed an exam and the potential consequences of this decision for this student’s future patients.

R6: *“I’m worried that somehow I had to allow them to pass this subject because it was not their fault […] I can see that they have very specific shortcomings, and in a few months, they will start working as midwives, and I’m sure that they are just not prepared.”*

This situation was also very emotional for some respondents and constituted a source of distress and empathy toward students.

R1: *“I mean, I feel sorry for them that their chances of working with the patient are limited, that we cannot perform any more of these procedures.”*

R20: *“Very strong emotions. Because everyone was afraid of the disease, infection, but most of all, when it comes to the role of the teacher, we were terrified of how we would teach these students, right? If we cannot show them, if they cannot perform these skills, then what will they be qualified to do later? And there was a lot of emotions here, a lot of discussions.”*

But at the same time, the feeling of one’s duty and obligation to others seems to become crucial in adapting to these new circumstances and putting additional effort into learning new skills, finding alternative ways to work, and managing time effectively.

### Building task-specific self-efficacy

Feeling overwhelmed by the multitude and variety of tasks entrusted to them, some of the respondents admitted to presenting resistance towards this change or a temptation to procrastinate.

R8: *“Initially there was this feeling of resistance that it was impossible, it was pointless, but when we started to show that we cannot otherwise because we will not reschedule these classes, nor are we able to, I don’t know, to cancel them. Well, they have to take place. Because in March, at the beginning of the pandemic, there were voices that it would be 2–3 months and we would go back to what was before, so there was a temptation to postpone some of the classes to June, maybe part of July, so there was a moment, a bit of an escape from it […].”*

R24: *“I remember that at the beginning, the attitude towards these online classes was full of reservations. And at the beginning, we were looking for some ideas on how to go around the didactics. […] it was not known how much we should prepare for these online classes, how long will it take, whether we should put a lot of energy into it.”*

However, when it turned out that the crisis would last for a longer period of time, clinical teachers began to gradually shift their focus on the specific tasks they faced and look for solutions that would enable their effective implementation in the new reality. One of the key elements at this stage turned out to be institutional support visible through securing and providing tools allowing maintaining the continuity of the teaching process. Those respondents who saw support from the University provided some examples, which mostly revolved around the availability of didactic resources, courses, technical issues, and reduced teaching hours in justified cases.

R13: *“I think the University tried to react very quickly and adapt as quickly as it could. […] Practically overnight, these tools were made available for work, and as we know earlier, it was not like that that the entire University worked on a huge messenger application [Teams].”*

R11: *“It must be admitted that it was known how to act and what to do. […] Personal protective equipment, masks, gloves, and disinfectants were also provided.”*

R25:* “There were certainly prepared aids about Teams—how to use this platform […] if something did not work, the IT team would surely help us, and we knew we could count on it.”*

As a result of difficulties in conducting clinical classes, teachers were forced to look for substitute solutions to facilitate students’ learning of practical skills. An important solution provided by PUMS decision-makers was access to the Medical Simulation Center, which had an important impact on enhancing their self-efficacy in terms of teaching practical skills but also motivating students to pay bigger attention during online classes.

R2: *“And due to the fact that I have the possibility, in addition to conducting e-learning seminars, I prefer to transfer it all later into a simulation center—it leads to their bigger interest in what I say because they know they will be able to see it. They know that when I say something, I will verify it in the practical part.”*

Despite these interventions, one of the commonly mentioned factors that could have reduced teachers' self-efficacy was the occurrence of technical and Internet connection issues, insufficient equipment amounts, or inappropriate conditions to conduct classes.

R5: *“I have observed it even at my clinic—I have my own room that I work in, I have my computer, I can close [the door], hang a piece of paper – ‘I have classes, please do not disturb.’ But what can an assistant do sitting in a room with three other assistants? […] through the prism of my friend – ‘listen, what should I do? I don’t have conditions, there is no library’, there is no room to find there so that she could do as I do. So, my friend had to go home to have classes with students so that it would take place in comfortable conditions. And then she had to return to work - to her other duties.”*

R23: *“We are not prepared for the number of online classes in hospitals. I mean, we use our equipment, we use our personal computers, webcams, we don’t have rooms where we could hide for these classes and conduct them during work, right? Because we are in the hospital at that time and we have to hide somewhere. Two people who lead Teams [classes] next to each other, well, you can’t conduct classes like that. So, this is also an organizational problem for sure. […] Some doctors had a problem that they didn’t have the equipment in order to run these classes, or the Internet – not everywhere is the Internet good enough to conduct these classes.”*

Another important factor that affects teachers’ self-efficacy in the context of clinical education was their clinical experience. While more experienced clinicians, having the freedom to use the rich base of resources accumulated so far, could concentrate on reorganizing their use in the course of classes, at the same time, the young clinicians had to search for or even create the necessary resources from scratch.

R4: *“It often happened when we talked about depression because in this theoretical part, I, for example, simulated a depressive patient. […] And we role-played such scenes, what if during the whole interview, the patient, for example, cries, what if he screams, what if, I don’t know, he insults, and we formulated these questions depending on the situation. It required a lot of my commitment as an assistant and a lot of clinical experience. Because, let’s not hide, after 18 years of work and thousands of patients, I can play every patient at the click of a finger. […] It was certainly more difficult for assistants who, for example, were only starting their teaching work because they had fewer resources they could draw from in the context of experience.”*

Among the factors that respondents perceived as important for rebuilding their efficacy in the role of an academic teacher was their previous participation in faculty-development (FD) initiatives. Some respondents acknowledged that they facilitated the transfer of practical teaching into the online environment. The knowledge and awareness of available solutions gave them a sense of control over the changing situation and thus increased their self-efficacy.

R6: *“[talking about the FD course] Well, our trainer showed different forms of education, but it was rather as a curiosity, also e-learning, but it was rather just as an additional area, that there are such opportunities, that some universities also conduct classes in this way if the students are from away, for example, that it makes it easier in a certain way and she just showed us various instructional videos and various tools, these quizzes, for example—how to motivate students for example. Thanks to this, I was not afraid to start doing it, although I was not really prepared for it.”*

The issue of evaluating students' qualifications remained an unresolved problem affecting the sense of self-efficacy of teachers. Online assessment in the form of, so far commonly used, test exams was considered unreliable (in regard to students’ cheating and their use of books, notes, or the Internet during test exams). Meanwhile, replacing them with theoretical oral exams was too time-consuming, and access to practical exams was severely limited due to the pandemic restrictions. Some respondents questioned the quality of the didactic process carried out using online methods, so, not wanting to harm students, they avoided verifying students' higher-order cognitive skills during the assessment.

R23: *“The reliability of assessment was certainly better before the pandemic. And it certainly was a more complete assessment because now I can ask them about a few things, and in fact, not having the feeling that I could teach them something well, it is hard to question them about something difficult or more advanced because I do not have the feeling that this teaching has any level, to be honest. And I would not like for them simply to get a worse grade as a punishment for the pandemic. So, I think that this reliability is worse because they do not have the knowledge, and I don’t have the conscience to question them on the lack of knowledge caused after all not by us, in fact, rather by the world.”*

### Development of general self-efficacy

In view of the pandemic-enforced limitations on students’ presence in hospitals in clinical classes, some teachers were forced to draw a clear line between their duties as a teacher and, separately, as a clinician and, as a consequence, to prioritize their duties. Respecting the complexity of the situation of clinical teachers and introducing solutions at the institutional level gave the respondents a sense of security and clearly influenced their self-efficacy.

R10: *“[The support from the University] was, in my opinion, big. Firstly, the possibility that we didn't have to come to classes at times when we had absolutely no way to come [due to clinical work]. Secondly, there was a reduced teaching load for people working in the COVID hospital […] there was a moment when I was afraid that they [clinical teachers] would not come to these exercises, and then what? And when the University announced these changes, many people were relieved it could be combined. We had no way to come back from the COVID hospital. We couldn't drop everything and go teach. And we would be in a pickle if the University hadn't made some moves here.*”

Many of the respondents' statements indicate that care for patients occupied a higher place in the hierarchy of tasks than the implementation of the didactic process (see section *Task-specific self-efficacy* quotes R8 and R24). The separation of these roles gave some teachers the freedom to conduct classes in comfortable conditions without the feeling of neglecting their tasks in the other role.

R19: *“During clinical classes in clinical hospitals, there is very little space dedicated to students, classrooms where you can sit down quietly, discuss certain things, so the online classes, when I sat in front of the computer in the office, and I didn’t have to think about or look for a classroom for students, they were kind of a facilitation.”*

Although seemingly the possibility of finding time dedicated only to students should be conducive to building a partner relationship between teacher and learner in the educational process, online learning during the pandemic restricted interpersonal contact in an unprecedented way. Respondents noticed that students became more passive and started to hide behind the cameras. They reported difficulties initiating contact with them, getting to know them, interpreting their behavior, getting feedback on their teaching, or even convincing them to turn on their cameras. This lack of interaction with students translated into teachers’ lower sense of effectiveness and satisfaction from conducting classes.

R6: *“I can’t cope with the situation when students don’t want to turn on the camera—I tell them that it would be nice because it’s hard to talk to the black screen, but if someone doesn’t do it, ignores me—I don’t know what to do then. Theoretically, of course, I can say that they will not pass the classes, but this is putting it on edge.”*

*R23: “I am worn out. Because, in general, I can see that it does not fall on a [fertile] ground, in the sense that nobody wants to—they don’t want to, I don’t really want to because I know that it doesn’t bring anything. I’m trying, right? Well, because everyone is trying […] I’m generally tired, I’m tired because this is just talking, there is no such interaction, they have no questions […] after these classes, I am generally tired and depleted of strength.”*

This lack of direct contact with students was especially difficult for some teachers, who get emotionally attached to students.

R2: *“Well, there was sometimes anger that I can’t do that, that I can’t get in, that I have to do e-learning classes, that I can’t show, meet these students. I am more focused on contact with students when I see them, see their reactions, emotions, I am with them rather than the cameras turned off and talking to the laptop.”*

One respondent also expressed a wish for more feedback on her teaching, given the new and extraordinary character of the situation, as well as reduced feedback from other sources, e.g., students. It accounts for the lack of confidence in non-contact classes.

R6: “*I have a lot of doubts about the quality of my classes conducted online when this subject requires contact with the patient and quality and effectiveness. I cannot even judge at all if this is it—these are the classes where I give lectures and seminars. Because for me, such personal contact with another person is very important because then some relationship is established to feel the specific energy of this group of students, I am completely unable to find myself when these classes are only in the online form. I don’t know how they perceive it at all. […] I need someone to tell me if I’m doing it right and if I’m not doing it right, then how to do it better […] I asked for auditing of these classes […] I don’t know if I designed it, planned it well, or if the tasks I gave are OK.*”

Similarly important for teachers’ self-efficacy was the presence or lack of support from their co-workers. The uplifting importance of a sense of community was emphasized at many different stages of realizing the professional tasks. This support could take the forms of both contact groups for members of the faculty and individual interactions between the teachers.

R4: *“I say a support group, that is, as we did groups, individual subgroups for students, I also created so-called assistant groups […]. It was also cool because it was a place where you could get help quickly, and we also saw what other assistants were doing so that it was at the same level for all students. […] Someone, for example, attached some material – ‘I have a proposal, maybe we could include it? It could be done so and so’—and everyone accepted it, and then very quickly on the same day, it was possible to add or modify something for the students.”*

R5: *“Very good, we supported, we talked, it wasn’t like that that I thought of a way on an ongoing basis, but then we talked with my friend – ‘I did it so and so, and how do you do it?’—and we collected what could be done better to make it more creative. Well, he said – ‘I will also be showing some endoscopic pictures’ – we all were drawing one from another. I think these relations were very good. We were all supporting each other.”*

R21: *“I think that such group therapy and group support played a large role here, in the sense that we discussed various topics, we tried to find common solutions, and all these discussions about our internal fears somewhere, that we were on the contagious ward, there was a higher probability that we would get infected in the hospital and transfer it to our relatives and so on. So, I think we’ve been a lot of support for each other in these difficult times, and somehow as a team, I think we passed the exam, and we did it.”*

Despite the online learning limitations described above, the COVID-19 pandemic has contributed to the dynamic development of educational technologies, giving teachers unprecedented opportunities to support the educational process. Some respondents emphasized that the widespread access to these tools allows them to better moderate the learning process.

R8: *“Contrary to appearances, I discovered that on Teams, I was able to involve each individual student more—I discovered that. It was incredible for me because when each of them had their homework, each of them had to confront me, share their thoughts […] So this is the thing that seemed to be better at Teams. I discovered it because I tried to involve everyone during my clinical classes, but I never felt that I was able to engage everyone in 100%. And here they had no choice—everyone had a task, everyone had to solve it.”*

On the other hand, for some respondents, the pandemic also brought positively perceived changes in the specifics of their scope of practice or performed tasks. For example, pharmacists observed legal recognition of the long-awaited expansion of their competencies, while physicians were provided with self-development opportunities by caring for patients outside of their specialty.

R17: *“I think it changed a lot in the context of my responsibilities and what is really going on in the pharmacy because even vaccinations or changes to the Act on the Pharmacist’s Profession—all of this really happened due to the pandemic, thanks to the pandemic.”*

R23: *“I learned a lot of new things. We became a COVID ward, and I had to learn everything really—from diabetes to leukemia, so I think it had developmental effects on us. It mainly increased our development and the profile of those [patient] cases that we had.”*

## Discussion

Previous studies confirmed the applicability of Bandura’s conceptualization of self-efficacy in research on teacher efficacy [31]. This self-efficacy constitutes a cognitive process during which people, in this case, the teachers, build their beliefs in their capabilities to manage certain situations, which in turn affect the amount of effort put into handling them and the response to any failures or obstacles that arise in the meantime, including persistence, resilience and experienced stress [17, 32]. Unfortunately, only a few studies on factors associated with teachers’ self-efficacy conducted so far concerned medical teachers.

In view of limited reports on the self-efficacy of medical teachers, and taking into account the growing disproportion between the demand and supply of services provided by qualified medical personnel, resulting in an increasing work-overload among them, and since according to Tschannen-Moran and Hoy [33], *“efficacy beliefs influence teachers’ persistence when things do not go smoothly and their resilience in the face of setbacks,”* this study aimed to identify factors affecting self-efficacy of medical teachers during the crisis caused by the COVID-19 pandemic.

Although some of the factors seem consistent with those described in the literature for classroom teachers, the obtained results clearly indicate that the observed decline described by the respondents was closely related to their many roles in their daily professional duties. Tschannen-Moran et al. [32] stated that *“studies of efficacy […] tend to focus on the knowledge and beliefs of teachers and not on the cultural meaning of efficacy in terms of the roles, expectations, and social relations that are important in the construction of those teacher beliefs”*. Chen et al. [34] even distinguished a category regarding the environment. In this work, particular attention has been paid to characterizing the experience of clinical teachers and building an understanding of the elements unique to the above-mentioned professional group of medical teachers, with regard to whom roles, expectations, and social relations differ from those related to classroom teachers.

Crucial to this project is a clear link between the identified factors and sources of self-efficacy identified by Albert Bandura, namely enactive mastery experiences, vicarious experiences, verbal persuasion along with other social influence types, and physiological and affective states [21], which is presented in Table 3 and discussed in detail below.

**Table 3:**
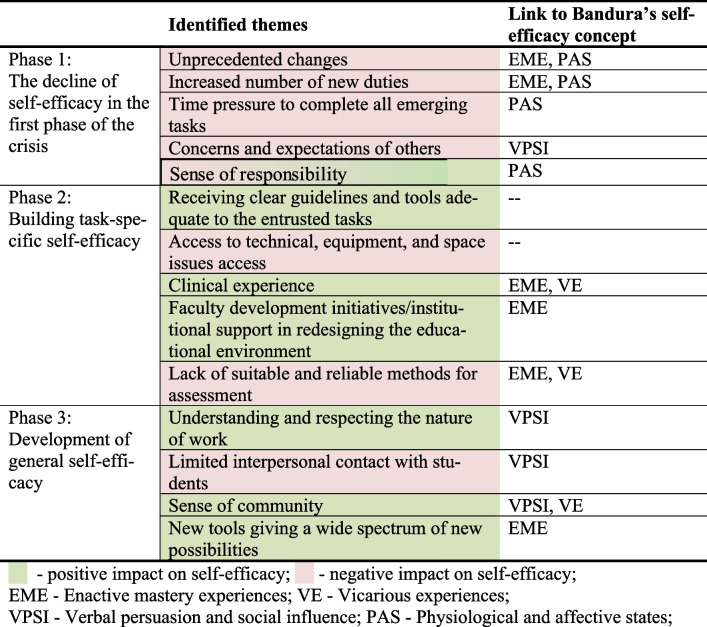
Factors influencing clinical teachers’ self-efficacy at different stages of health crises

### Enactive mastery experiences

Mastery experiences represent the upbuilding effect of successes and the deteriorating effect of failures on one’s self-efficacy beliefs. In this context of one’s previous experiences, it should be noticed that the outbreak of the COVID-19 pandemic was sudden and unexpected. Similarly to most of the other teachers, Polish clinical teachers were never before in a situation where the entire learning process had to be moved to online conditions. Simultaneously, unprecedented changes also involved their other professional role as clinicians—working in such conditions was a complete novelty for them. Therefore, in view of the need to develop completely new work standards, we observe a complete collapse of their sense of self-efficacy in the first phase of the crisis. However, obstacles may also serve as learning opportunities to overcome them [21]. In the context of our study, the COVID-19 pandemic and the identified challenges associated with the clinical teaching environment and limited time to manage all duties might be viewed as such obstacles. In response to the crisis, clinical teachers began to familiarize themselves with new methods and tools they had never used before. The way in which medical teachers were able to overcome, for instance, difficulties associated with work overload or changes in their clinical and didactic environment, find substitute solutions for clinical skills learning during the pandemic, or overcome technological difficulties seemed to positively influence their self-efficacy. The experiences described by the respondents seem consistent with the Dreyfus Model of Acquisition, according to which, when acquiring new skills, the individual passes through five stages, namely: novice, competence, proficiency, expertise, and mastery [35]. Teachers who initially followed the guidelines and used the technologies provided by PUMS as "the only chance" to conduct the planned classes gradually gained experience in using them and, after some time, started to consciously include elements of distance education and simulation into the regular learning process, striving to increase the effectiveness of the designed learning experiences.

Similarly, not without significance was also the role of their previous clinical and teaching experience. In a study by Yang et al. [36], teaching seniority and teacher qualifications were among the factors influencing clinical nursing teachers’ job self-efficacy and involvement next to their age, marital status, and job satisfaction. In our study, in the absence of access to patients, experienced teachers were more easily able to recall a wide range of previous clinical experiences, enabling students to analyze a wide variety of clinical cases.

There also seems to be a relation between teachers’ and students’ sense of efficacy [33]. For teachers, mastery experiences can be formed when they observe improvement in their students’ performance. In the presented study, the respondents not only pointed to the difficulties with conducting a reliable assessment of students’ qualifications but also directly admitted that during the assessment process, they tended to lower expectations towards students, bearing in mind the deteriorating effect of the pandemic on the quality of the educational process. The collected data seem to confirm the results of Dybowski et al. [37], which showed teachers’ self-efficacy seems to be a predictor of their perceptions of students’ competencies. Therefore, it seems critical to help teachers succeed in this way by providing them, for instance, with sufficient support and resources in the initial phases of online teaching [38]. Also, in a failure situation, whether a teacher is willing to stay with a student may serve as an indicator of their teaching self-efficacy or students’ learning ability [31]. A study conducted by Chen and Yeung [34] on Chinese teachers in Australia revealed three categories of factors affecting teachers’ self-efficacy, namely teacher, student, and contextual factors. In the context of mastery experiences, teacher factors involved, among others, aspects of professional learning, teaching experience, and understanding of their students, while student factors included students’ responses, discipline in the classroom, students’ motivation, or their relations with the teacher, for example [34]. In the COVID-19 context, this all leads to a conclusion that to provide effective teaching in times of crisis, students should be even stronger motivated to take responsibility for the educational process. The involvement of students facilitates the involvement of teachers in conducting classes. This beneficial effect on teachers’ self-efficacy was also confirmed by Raudenbush et al. [39]*,* stating that *“low-track classes present challenges to teachers that make it difficult for them to maintain elevated perceptions of self-efficacy.”* Although they studied high school teachers, it is worth noting that this relationship was particularly visible among math and science teachers. Unfortunately, in the COVID-19 context, besides technological issues, also student motivation was perceived as a significant challenge to online medical education [40].

### Vicarious experiences

In turn, vicarious experiences serve as a point of reference and draw from observation and comparison of one’s capabilities with other people [21]. In the context of medical and medical teachers’ self-efficacy, the available literature pays much attention to the issue of adequate institutional support and faculty development. Sethi et al. [41] evaluated the effect of a renowned international postgraduate program showing its positive effect on teachers’ development and behaviors in medical education, including their self-efficacy and sense of belonging to the educational community. Similarly, a positive impact of a longitudinal faculty development program on the self-efficacy of health professions teachers was evidenced by Singh et al. [42]. Finally, Tenzin et al. [43] also observed an increase in participants’ self-efficacy and teaching competencies due to the faculty development program. However, they also listed inadequate support from the relevant decision- and policy-makers and the lack of a medical education center among important challenges in their implementation. Meanwhile, effective faculty development interventions may also take simple and cost-effective forms and still be helpful and well-received by potential recipients [44]. Our respondents also paid attention to the role of support and consultations with co-workers or faculty development initiatives. In the crisis situation, such as the current pandemic, they seemed to provide teachers with a sense of stability and control. According to Raudenbush et al. [39]*, “teachers’ increased control over their working conditions and increased opportunities for collaboration with other teachers can enhance their perceived self-efficacy.”* The term community of practice was coined to denote *“groups of people who engage in a process of collective learning in a shared domain of human endeavour”* [45]. Although regular interactions are essential, online communities of practice can also be achieved with the use of modern technologies [46]*.* Ekici et al. [46] showed that online communities of practice could significantly improve teachers’ self-efficacy by providing teachers with such benefits in terms of vicarious experiences as possibilities for sharing, comparisons, realizing their own weak points, and drawing from others’ experiences, for instance. Not without significance is also the sense of technological self-efficacy of the teachers.

### Verbal persuasion, along with other social influence types

Verbal or social persuasion underlines the importance of the belief of significant others in one’s capabilities and positive feedback [21]. For teachers, their self-efficacy may be influenced by reactions from students and co-workers [34], which was also mirrored in our study. While lowered motivation and attention of students were viewed as obstacles and disadvantages of online learning, teachers often referred to their co-workers from their departments as an important source of social interactions during the pandemic. Individual feedback or verbal encouragement can also be provided as a self-efficacy boost, for instance, from co-workers or supervisors, when a new teaching strategy is being implemented [47]. Due to the novelty of online clinical education and hindered interpersonal contact with students, our respondents were receiving little student feedback on their teaching, which seemed to lower their self-efficacy. Similarly, one teacher also suggested bigger involvement of the University in providing teachers with feedback in these unprecedented circumstances. Following Bandura’s theory, in this context, the self-efficacy improvement could be achieved, for instance, in the form of continuous feedback where new skills are learned, put into practice, and their implementation provided with feedback [48]*.* Additionally, an interesting example of the importance of significant others’ belief in the context of the clinical role of the teacher was provided by the legal recognition of the expansion of pharmacists’ competencies introduced by the governmental authorities and its positive reception among those teachers who are pharmacists. This seems consistent with previous reports showing the readiness and preparedness of members of this profession to perform other tasks during the pandemic [49], as well as the impeding role of the absence of appropriate legal regulations on broadening the scope of practice [50].

### Physiological and affective states

Finally, the physiological and emotional states also can affect one’s self-efficacy [21]. The stress, anxiety, uncertainty, and fatigue accompanying the COVID-19 pandemic in their everyday life or resulting from, among others, work overload, frequent restriction changes, and the limited possibility to convey practical skills were examples frequently mentioned by the respondents. In the study by von Muenchhausen et al. [19], the self-efficacy of teachers was connected with their work-related psychological resistance and positive emotions, with its increase associated with improved life satisfaction and distancing ability and a decrease with lowered social support experience. There also seems to be a strong connection between the self-efficacy of teachers and their innovative behavior [51, 52]. In the case of our respondents, it also seemed to work the other way around since the creativity to find alternative solutions for practical learning increased their morale and sense of self-efficacy. Teachers’ self-efficacy also seems to positively and interactively correlate with their empowerment [53]. Meanwhile, as a result of the significant work overload, the balance of the teaching, scientific activity, and clinical practice in the aforementioned three-legged stool reference [1] was seriously disturbed, leading to a sense of pressure of responsibility for patients and students, which the respondents manifested in the interviews. Even though task-specific self-efficacy is not theoretically related to global self-efficacy, still, some respondents indicated that being suddenly overwhelmed from so many different sides triggered a feeling of general powerlessness and depletion.

### Access to technology and space to conduct the classes

The obtained results also indicate that ongoing technological development increases our dependence on technology in everyday and professional life. This phenomenon was emphasized during the pandemic and the resulting transition into distance learning. As it was visible in the statements of our respondents, whether they had the conditions to conduct online classes also seemed to influence their self-efficacy. It should be emphasized that this issue seems separate from the technological skills and self-efficacy in using it, which were covered in the section dedicated to enactive mastery experiences. However, the specificity of clinical teachers’ work creates several conditions that may be unique for this group of teachers. For example, as presented in respondents’ statements, unlike most other teachers who, during the lockdown period, were conducting classes from their homes, some clinical teachers had to conduct their classes from the hospitals. This led to unique difficulties like an inadequate number of computers on the ward or even limited space to conduct the classes due to the number of co-workers in one doctor’s office. Although we were not able to attribute this issue to any of Bandura’s sources of self-efficacy in Table 3, we believe that it deserves attention. Firstly, we recognize that when the concept of sources of self-efficacy was developed, the human dependence on technology was lower. Secondly, it also seems to originate in the combination of two highly specific and unique conditions, namely the lockdown and distance learning during the COVID-19 pandemic and the specificity of clinical teachers’ work. Therefore, while many authors strive for a strict distinction between pedagogy and technology, this can lead to a false dichotomy [54]. Contemporary teachers, including clinical teachers, should be aware of the inseparable relationship between pedagogy and technology because using the latter in the educational process is not only a medium for the former. They rather intertwine in the form of mutual shaping of purpose, context, values, and methods.

### Limitations

Our results should be interpreted within the limitations of the study. This was a single-center study, and teachers from other universities could have different perspectives on the topic. However, taking into account the qualitative nature of the research and the identified knowledge gap in the field of the discussed issues, we believe that the collected data may be relevant and useful for decision-makers in the field of medical education. Moreover, questions have been raised about *“the extent to which teacher efficacy is specific to given contexts and to what extent efficacy beliefs are transferable across contexts”* [33]*.* Nevertheless, even if we cannot generalize these results beyond the pandemic period, insights into factors potentially affecting medical teachers’ self-efficacy during a health crisis may have an impact on both the quality of their didactic and health services, which needs to be confirmed in further research. Furthermore, to increase the credibility and trustworthiness of this study, we decided to use researcher triangulation during the data analysis and follow standards for reporting qualitative research.

## Conclusions

Some of the identified factors coincide with those influencing the self-efficacy of other teachers. However, as our study shows, it is important to provide medical teachers with special care during a health crisis, as the lack of an appropriate response from decision-makers may have long-term consequences. The obtained results can be used by hospital and university authorities to better understand the impact of the COVID-19 pandemic and any similar future events on the self-efficacy of clinical teachers and help them to provide support for the teachers and better respond to the identified problems. The quantitative assessment of the sense of self-efficacy among medical teachers requires the creation of a dedicated quantitative tool that, on the item list, should take into account the specificity and context of work performed by members of this professional group. Decision-makers responsible for crisis management on the part of educational institutions should consider in their strategies the clinical involvement and the resulting burden on clinicians. Similarly, when making staff decisions, healthcare facilities’ management should also consider the amount of work resulting from other duties of medical teachers. The organizational culture of medical universities should involve faculty development initiatives and foster a sense of community of practice and teamwork. There is also a need for further research on the correlations between teaching practices, outcomes of the students, and the self-efficacy of teachers.

## Data Availability

The datasets used and/or analyzed during the current study are available from the corresponding author on reasonable request.

## Abbreviation

PUMS: Poznan University of Medical Sciences
